# Inhaled Exosomes Genetically Manipulated to Overexpress CD24 (EXO-CD24) as a Compassionate Use in Severe ARDS Patients

**DOI:** 10.3390/biomedicines11092523

**Published:** 2023-09-13

**Authors:** Orr Green, Gil Shenberg, Roni Baruch, Lihi Argaman, Talya Levin, Ian Michelson, Ruthy Hadary, Boris Isakovich, Miri Golos, Reut Schwartz, Ronan MacLoughlin, Nimrod Adi, Nadir Arber, Shiran Shapira

**Affiliations:** 1 Health Promotion Center and Integrated Cancer Prevention Center, Tel-Aviv Sourasky Medical Center, Tel Aviv 6423906, Israel; orrgreen@gmail.com (O.G.); gils@tlvmc.gov.il (G.S.); lihibz@tlvmc.gov.il (L.A.); ianstl8@gmail.com (I.M.); narber@tauex.tau.ac.il (N.A.); 2 Sackler Faculty of Medicine, Tel Aviv University, Tel Aviv 6423906, Israel; roniba@tlvmc.gov.il (R.B.); reutschvartz@gmsil.com (R.S.); nimroda@tlvmc.gov.il (N.A.); 3 Department of Kidney Transplantation, Tel-Aviv Sourasky Medical Center, Tel Aviv 6423906, Israel; 4 Department of Internal Medicine C, Meir Medical Center, Kefar-Saba 4428164, Israel; ruhadary@gmail.com; 5 Intensive Care Unit, Hillel Yaffe Medical Center, Hadera 3820302, Israel; borisi@hymc.gov.il; 6 Carmel Medical Center, Haifa 3436212, Israel; mirigolos@clalit.org.il; 7 Anesthesia and Intensive Care Unit, Tel-Aviv Sourasky Medical Center, Tel Aviv 6423906, Israel; 8 School of Pharmacy and Biomolecular Sciences, Royal College of Surgeons, D02 YN77 Dublin, Ireland; rmacloughlin@aerogen.com; 9 School of Pharmacy and Pharmaceutical Sciences, Trinity College, D02 PN40 Dublin, Ireland

**Keywords:** exosomes, CD24, EXO-CD24, acute respiratory distress syndrome, ARDS, COVID-19, inhalation, nebulizer

## Abstract

Rationale: Acute respiratory distress syndrome (ARDS) is a major global health concern with a significant unmet need. EXO-CD24 is delivered via inhalation-reduced cytokines and chemokine secretion and lung injury in ARDS and improved survival in mice models of ARDS, influenza, and sepsis. Objectives: This clinical paper aims to evaluate the potential of EXO-CD24, a novel immunomodulatory treatment, in the compassionate care of critically ill, intubated patients with post-infection-induced acute respiratory distress syndrome (ARDS). Methods: Eleven critically ill patients diagnosed with post-infection ARDS (10 with COVID-19 and one with an adenovirus-associated infection) were administered EXO-CD24 in four medical centers across Israel. The patients had multiple co-morbidities, including cancer, hypertension, diabetes, and ischemic heart disease, and met the criteria for severe ARDS according to the Berlin classification. EXO-CD24 was administered via inhalation, and adverse events related to its use were carefully monitored. Measurements and Main Results: The administration of EXO-CD24 did not result in any recorded adverse events. The median hospitalization duration was 11.5 days, and the overall mortality rate was 36%. Notably, patients treated at the Tel Aviv Medical Center (TASMC) showed a lower mortality rate of 12.5%. The WBC and CRP levels decreased in comparison to baseline levels at hospitalization, and rapid responses occurred even in patients with kidney transplants who were off the ventilator within a few days and discharged shortly thereafter. The production of cytokines and chemokines was significantly suppressed in all patients, including those who died. Among the patients at TASMC, four had kidney transplants and were on immunosuppressive drugs, and all of them fully recovered and were discharged from the hospital. Conclusions: EXO-CD24 holds promise as a potential therapeutic agent for all stages of ARDS, even in severe intubated cases. Importantly, EXO-CD24 demonstrated a favorable safety profile without any apparent side effects with promising efficacy. Furthermore, the potential of EXO-CD24 as a platform for addressing hyper-inflammatory states warrants exploration. Further research and larger-scale clinical trials are warranted to validate these preliminary findings.

## 1. Introduction

Acute Respiratory Distress Syndrome (ARDS) is a clinical syndrome of acute respiratory failure due to diffuse lung inflammation and edema [1,2]. ARDS is a major health concern with 3 million new cases annually. This life-threatening disease is characterized by vascular leakage leading to severe hypoxemia and often necessitates invasive mechanical ventilation. ARDS is associated with elevated morbidity and mortality rates, resulting in the loss of 1.2 million lives annually on a global scale. Rapid clinical deterioration can manifest over a period of 24 h [3].

There is no medical therapy for ARDS [4]. A lung protective strategy of mechanical ventilation remains the only disease-specific therapy shown to improve survival [4]. The treatment in ARDS gained further momentum during the COVID-19 pandemic. About 5% of individuals with COVID-19 pneumonia may develop a hyper-inflammatory state, which can in turn lead to ARDS [5].

A cytokine release storm (CRS) is the most severe complication of ARDS that leads to respiratory failure. CSR is a hyperactive immune response characterized by the release of pro-inflammatory mediators that is disproportional and increases to a deleterious level in the patient [6,7]. Early diagnosis and therapy to suppress the CRS may contribute to the prevention of respiratory failure and a disastrous outcome.

As such, a therapeutic regimen that would dampen unwarranted immune processes, while not interfering with anti-viral immunity and can be applied to other diseases, is of significant interest. 

Cluster Differentiation 24 (CD24) is a small, heavily glycosylated Glycosylphosphatidylinositol (GPI)-anchored protein which serves as a dominant innate immune checkpoint, in other words, a “do not eat me signal”. CD24 plays an important role in controlling the homeostatic proliferation of T cells; hence, it can negatively regulate inflammation [8]. Recently, a recombinant fusion protein composed of the extracellular domain of CD24 linked to a human immunoglobulin G1 (IgG1) Fc domain has been identified as a potential immune checkpoint inhibitor with anti-inflammatory activity [9]. This protein plays a crucial role in facilitating immune differentiation between Damage Associated Molecular Patterns (DAMPs), which are released from damaged or dying cells, and Pathogen Associated Molecular Patterns (PAMPs). Hence, CD24 helps in regulating immune response, preventing excessive activation while still allowing the effective clearance of pathogens [10,11,12].

CD24, Siglec 10, and HMGB1 form a trimolecular complex that exerts negative regulation on the NF-kB pathway [10,13,14]. This complex allows for the discrimination between PAMPs and DAMPs, enabling the differentiation of “self” from “non-self”, such as bacteria and viruses. CD24, in particular, binds to DAMPs, preventing their interaction with pattern recognition receptors (TLRs) and further inhibiting the activation of the NF-kB pathway [11,12,13]. This dual immune checkpoint inhibition demonstrates anti-inflammatory activity. The advantage of CD24 over steroids lies in its ability to selectively modulate the host response to DAMPs without interfering with PAMPs’ recognition, which is crucial for an effective immune response.

Exosomes are intraluminal nano-vesicles that play a vital role in intercellular communication [15]. They are currently being investigated for their potential as diagnostic tools and carriers of therapeutic agents in clinical research [16,17,18] One of the major benefits of exosomes is their high level of biocompatibility and low immunogenicity, due to their structural similarity to the cell membranes from which they originate [19]. Being endogenous vesicles, exosomes are not expected to stimulate the immune system or cause heightened side effects. Due to a small degree of manipulation needed to create exosomes, the characteristics of exosomes can be determined via known markers (CD63, CD81, and HSP70) [20,21,22,23].

We engineered EXO-CD24, which are exosomes genetically enriched with CD24 glycoproteins. EXO-CD24 was designed as a targeted therapy for hyperactive immune systems in the context of ARDS induced by COVID-19.

The delivery of therapeutics directly to the lung via the nebulizer is well established in the critical care setting and concurrent aerosol delivery during mechanical ventilation and other respiratory support interventions offers the potential for safe and effective administration without the need for further interventions, or indeed, the removal of the patient from the critical respiratory supports that they may have been prescribed [24]. The aerosol-mediated delivery of exosomes has already been shown to facilitate therapeutic outcomes and potential advantages over intravenous administration, where the activation of the complement system and the consequent risk of thrombus formation is a risk factor [25,26]. Indeed, in both clinical and pre-clinical models of a variety of diseases, the aerosol-mediated delivery of exosomes has already been investigated as a means of improving distribution throughout the airways, ranging from upper conducting airways down to the alveolar space. This ability to target areas of interest within the lung with high doses of exosomes has recently been shown to be effective also in the mitigation of Escherichia coli-induced pneumonia [27]. Nebulizers, specifically, have already been evaluated for the delivery of exosome formulations; however, to date, no regulatory approved exosome-nebulizer combination has yet been developed [28].

Further, given the complexity involved in optimizing the therapeutic formulation design for the storage in and administration by, e.g., pressurized metered dose inhalers and dry powder inhalers, nebulizers offer the ideal means of delivering simple therapeutic formulations containing high doses of the therapeutic exosomes to the ventilated, unconscious patient, without the need for complicated coordinated breath maneuvers on behalf of that patient. As was carried out in this study, exosomes can simply be suspended in physiologic saline and administered using an off-the-shelf nebulizer. Moreover, and importantly, nebulizers have been shown to be able to deliver via aerosol during lung protective ventilation strategies, which is a critical consideration in the care of the ARDS patient [24].

In a phase Ib/IIa study involving 35 patients with mild to moderate ARDS, the exceptional safety of EXO-CD24 was demonstrated without serious adverse events or even adverse effects related to the drug. Promising efficacy was noted as well [29,30]. A dose finding study (IIb) in 91 patients from three medical centers in Athens (Greece) confirmed the promising efficacy as compared to the controls and a lack of any side-effects related to EXO-CD24. Efficacy is currently being studied in an international, multi-center, randomized, quadri-blind clinical study of EXO-CD24 versus a placebo.

## 2. Materials and Methods

### 2.1. Cases Presentation

From March 2021 to November 2022, during the first and second phases of the randomized clinical trials, we encountered 11 patients with severe ARDS induced by COVID-19 that did not meet the inclusion and exclusion criteria of mild–moderate ARDS, as they were in a severe state of ARDS. The first eight patients were from TASMC, one patient was hospitalized during the first COVID-19 wave, and the rest in the second and third waves. The other three patients were from three hospitals across Israel (Carmel, Hillel Yaffe, and Meir). With the exception of one patient, all individuals were critically ill and intubated in an ICU. Four patients in TASMC had prior kidney transplantation procedures and were receiving immunosuppression drugs. The study was part of the compassionate-use program of the Israeli Minister of Health and was approved by the hospital IRB and the Israeli Minister of Health.

### 2.2. EXO-CD24 and Patients Follow-Up

The inhaled EXO-CD24 was administered via a standard jet nebulizer to these eleven critically ill patients with severe comorbidities once a day for five consecutive days. Two patients received the drug for 10 days and one patient received the drug twice a day.

0.5 mL of EXO-CD24 in a high dose (10^10^ exosomes), were diluted in 1.5 mL of saline, and administered via the nebulizer, using oxygen at 3–5 L/m for 4–5 min.

The routine monitoring was 24/7 throughout their hospitalization until they were discharged to other units. During drug administration, vitals were taken prior and following the inhalation. The patients had appropriate clinical follow-up as needed for 28 days, followed by monthly phone calls.

### 2.3. Human Multiplex ELISA Cytokine Array: Quantibody

Several-defined target analytes were chosen based on our previous experience in post COVID-19 ARDS patients [29]. The analysis was performed simultaneously using an array-based multiplex ELISA system. A combination of the high specificity and sensitivity ELISA was used with a high-throughput glass chip-based array. Briefly, a panel of capture antibodies was printed in multiple identical arrays on a standard slide. After a blocking step, the stored plasma samples were defrosted and incubated with the arrays. Nonspecific proteins were washed off, and the arrays were incubated with a cocktail of biotinylated detection antibodies, followed by a streptavidin-conjugated fluor. Signals were then visualized using a fluorescence laser scanner. Then, the RayBio Array Analysis Tool, an array-specific excel-based program, performed a sophisticated data analysis of the raw numerical data extracted from the array scan.

## 3. Results

There were eleven severely ill ARDS patients according to the Berlin Criteria, where 10 patients were diagnosed with COVID-19 and one patient with Adenovirus. The mean age was 68.7 ± 6.5, with 54.5% being men. A total of 45% of the patients had a history of smoking. The most common comorbidities were hypertension (HTN) (54.5%), ischemic heart disease (IHD) (45.5%), end-stage renal disease (ESRD) (36.3%), diabetes mellitus (27.3%), chronic obstructive pulmonary disease (COPD), or asthma (27.3%) (Table 1). There were two obese patients, one with an advanced stage of uterine cancer and one with lung adenocarcinoma.

During their hospitalization, different treatments were given according to the latest clinical updates throughout the pandemic. Aside from EXO-CD24, 54.6% were treated with Actemra, 81.8% were treated with dexamethasone, 36% were treated with remdesivir, and 18.2% were treated with Baricitinib (Table 2). It should be noted that all patients received adequate care, determined via innovative and well-considered decision-making without any interference from the research.

The median length of hospitalization was 11.5 days with a 35.4% mortality rate. Two patients (Meir hospital and TASMC) died in the five days of treatment, and two during the follow-up period up to 28 days at the Hillel Yafe and Carmel medical centers). The majority of the treated patients (63.6%) were discharged to their homes earlier than expected.

The laboratory exams were taken based on medical needs only (Table 3). In the surviving patients, the WBC and C-Reactive Protein (CRP) were decreased in 100% and 81.8% compared to baseline levels at hospitalization, respectively (Table 4 and Table 5).

To evaluate the reduction in pro-inflammatory cytokines and chemokines following EXO-CD24 treatment in more details, microarray studies (Quantibody Multiplex ELISA Array, RayBiotech, Almog Diagnostic Ltd., Shoham, Israel) were performed in patients’ plasma at time 0 (before treatment) and at the end of therapy.

**Figure 1:**
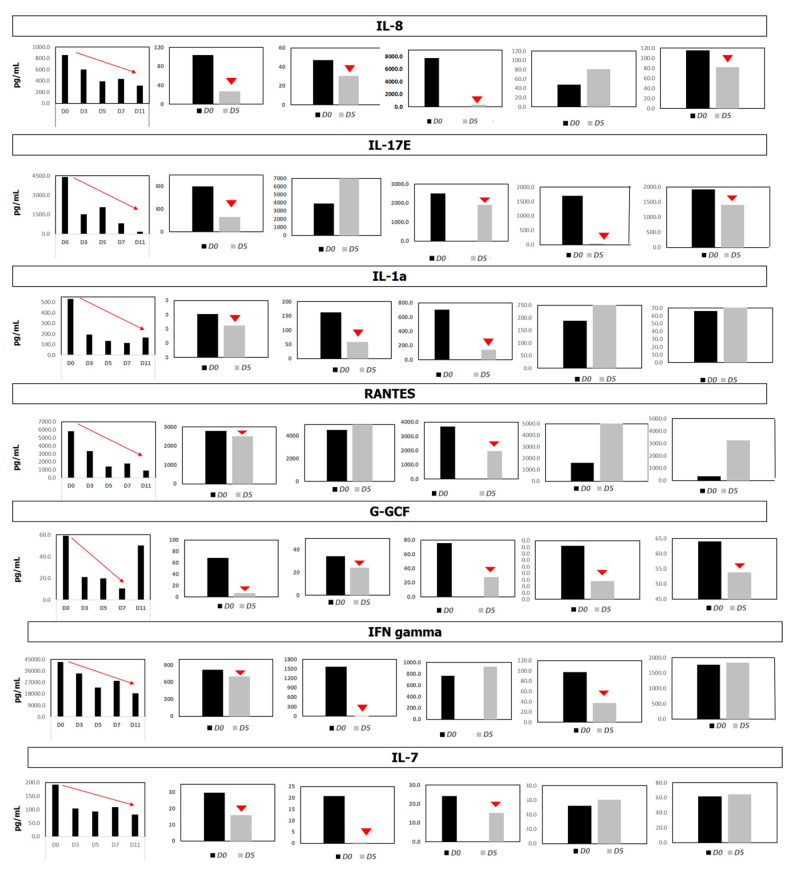
Cytokine levels in Quantibody Multiplex Elisa Array. Figure explanation: Systemic cytokine levels were measured before or after treatment with EXO-CD24 using Quantibody Multiplex ELISA Array. Cytokine and chemokines concentrations were quantified by comparing them to array-specific 8-point protein standards of each target protein. Representative results from six patients are shown.

A representative set of inflammatory indices, all known as NF-ĸB target genes, are shown in Figure 1 in six representative patients. Most of the tested analytes were significantly reduced at the end of therapy. Cytokine and chemokine concentrations were quantified by comparing them to array-specific 8-point protein standards of each target protein. The representative results from six patients are shown in Figure 1.

## 4. Discussion

EXO-CD24 delivered via the inhalation route suppresses the cytokine storm and may serve as a promising therapeutic agent in mild and moderate states of ARDS [11,12]. Herein, it is shown that EXO-CD24 can suppress the cytokine storm and may improve clinical outcomes and survival rates even in severe stage ARDS.

There are two breakthrough technologies that have contributed to this success, specifically the drug and the carrier. The drug CD24 functions in many signal transduction pathways. Most importantly, it acts as an immune checkpoint inhibitor and biological immunomodulator. It may serve as the new immunomodulator for many indications with an urgent unmet need. Exosomes are the ideal natural drug carriers. These nano-sized lipid vesicles are secreted by most cell types in the body, and play a critical role in intercellular communication [31,32]. EXO-CD24 exosomes are genetically manipulated to overexpress CD24 and are a combination of these two breakthrough technologies. 

The third important clinical achievement is the delivery route—inhalation. EXO-CD24 is delivered via inhalation directly into the target organ (the lungs). Aerosol-mediated delivery via inhalation allows for potentially non-invasive direct access to the lung and is suitable across the full range of patient types ranging across patient age, disease type, patient intervention, and patient interface. Appropriate aerosol device selection can influence critical determinants of success such as the total deposition of therapeutic agents and distribution within the lung [24].

EXO-CD24 can selectively attenuate DAMPs-initiated inflammation while allowing the body to mount a more robust response to PAMPs, and thus continue with an efficient pathogen clearance activity [11].

EXO-CD24 is a promising therapeutic agent not only for ARDS but may also serve as a breakthrough therapy for many diseases with an urgent, unmet need. For example, a proof of concept has been achieved in several animal models for abdominal (11) and pulmonary sepsis, pulmonary fibrosis, asthma, COPD, and influenza.

The treatment was administered to a total of 11 patients. The study comprised two groups: the first group consisted of four patients who underwent kidney transplantation, and the second group included seven patients with severe life-threatening ARDS. With the exception of one patient, all participants were ventilated in the ICU. No SAEs, or even AEs, related to EXO-CD24 were noted during the five treatment days and during follow-up for more than two years.

The mortality rate in ICU patients is very high at ~45% (Table 2). In TASMC, only one out of eight (12.5%) patients died within 48 h. The three other deaths occurred in patients that were treated in three different hospitals across Israel. In all of them, due to regulations and technicalities, therapy was initiated relatively late in the course of the disease as a last resource. A significant suppression of cytokine and chemokine production was achieved in all patients, including those who died. Most probably, lung injury was not reversable in these patients who died.

The ARDS post-COVID-19 was not related to the SARS-CoV-2 variant that the patients were infected with. It is important as EXO-CD24 has the ability to provide broad protection across variant types.

WBC and CRP were not in concordance with the clinical improvement. Leukocytosis in 75% of the patients was likely attributed to the use of steroids. Surprisingly, rapid responses were seen in the group of four patients with kidney transplants. They went off the ventilator within a few days and were discharged quickly thereafter. These surprising results may be thanks to a rapid identification of those patients and initiation of EXO-CD24 during their deterioration.

There were a few limitations, with the major one being an ethical consideration. No control group existed, as EXO-CD24 was given on a compassionate use basis. Another limitation was the lack of laboratory and metrics standardization, and the use in different medical centers and laboratories. CD24 has never been approved for clinical use, and exosomes only recently entered the clinical stage in several phase I-III studies. The potential efficacy stems from using the endogenous immunomodulator of the immune system, i.e., CD24. Safety is based on using other endogenous nanoparticle carrier exosomes that do not stimulate the immune system.

This study provides confidence in the non-invasive lung-targeted and topical administration of exosomes via aerosol in both ventilated and non-ventilated patients. Of note, separate studies investigating therapeutic delivery indicate that during ARDS-appropriate lung-protective low tidal volume ventilation, the expected dose delivered to the ventilated patient lung was just 5% of the nominal dose of exosomes placed in the jet nebulizer’s medication cup [33]. Similarly, in the single non-ventilated patient in this study cohort, during spontaneous breathing, it can be expected that the lung dose delivered via the jet nebulizer was also approximately 5% of the nominal dose placed in the nebulizer’s medication cup [34]. This further highlights the potency of EXO-CD24 when it was likely delivered to the lung in such low amounts.

Excitingly, those studies further highlight the significant potential for future improvements in the therapeutic application of exosomes with the adoption of high-performance vibrating mesh nebulizers, that, according to those same studies, facilitate the delivery of between four- and six-fold more drugs to the ventilated and non-ventilated patient lung respectively (~5% versus ~20+% in ventilated, and ~5% versus ~30+% in non-ventilated). This increase in the potential delivery of exosomes to the lungs allows for greater flexibility in treatment. The same dose, but now with more delivered to the lung, may provide greater benefit without the waste, or indeed, a smaller therapeutic dose may only be needed to be loaded into the nebulizer to achieve the same benefit. These are important variables in the development of novel therapeutics where the cost and complexity of manufacture are critical as well as the ability to control doses in a way that allows for the balance between efficacy and the potential for side effects. Furthermore, smaller nominal doses allow for more patients to be treated, and this is a key consideration in a pandemic response where often a limited supply of drug is a significant concern.

Finally, it is encouraging that the production of EXO-CD24 is effective, efficient, rapid, and at low cost, with a seamless technology transfer.

## 5. Conclusions

There is no medical therapy for ARDS, and EXO-CD24 may be the first therapeutic agent in all stages of ARDS. EXO-CD24 is a better immune modulator as it specifically suppresses DAMPs but not PAMPs and downregulates the cytokine storm without interfering with pathogen clearance. Efficacy must still to be confirmed in the clinical studies versus the placebo.

## Figures and Tables

**Table 1:** Patients’ medical history.

	Gender	Age	Diabetes	Obesity	Tobacco Use	ESRD	Post Renal Transplant	COPD/Asthma	IHD	CHF	HTN
Patient N. 1	M	64	0	0	0	0	0	0	0	0	0
Patient N. 2	M	72	0	0	1	1	0	1	0	0	1
Patient N. 3	F	73	0	0	1	0	0	0	1	0	0
Patient N. 4	M	77	1	0	0	0	0	1	1	1	1
Patient N. 5	M	34	0	0	0	1	1	0	0	0	0
Patient N. 6	M	76	1	0	0	0	1	0	0	0	1
Patient N. 7	F	71	0	0	0	1	1	0	1	1	1
Patient N. 8	F	69	0	1	1	1	1	0	1	0	1
Patient N. 9	M	73	0	1	1	0	0	1	0	0	1
Patient N. 10	F	67	0	0	0	0	0	0	0	0	0
Patient N. 11	F	78	1	0	1	1	0	0	1	1	0

**Table 2:** Treatment During Hospitalization.

	Remdesivir	Dexamethasone	Actemra	Bercetinib	Alive
Patient N. 1	0	0	1	0	1
Patient N. 2	0	1	0	0	1
Patient N. 3	0	0	1	0	1
Patient N. 4	0	1	1	0	0
Patient N. 5	1	1	0	0	1
Patient N. 6	1	1	1	0	1
Patient N. 7	0	1	1	0	1
Patient N. 8	0	1	1	0	0
Patient N. 9	0	1	0	0	0
Patient N. 10	1	1	0	1	0
Patient N. 11	1	1	0	1	0

**Table 3:** Serum Creatinine levels at hospitalization and during the drug administration.

Serum Creatinine Levels	Day of Hospitalization	Drug Administration Day 1	Drug Administration Day 2	Drug Administration Day 3	Drug Administration Day 4	Drug Administration Day 5
Patient N. 1	0.82	N/A	0.63	0.66	0.71	0.55
Patient N. 2	1.87	1.64	N/A	1.45	1.39	1.9
Patient N. 3	0.83	0.81	0.65	0.55	0.56	0.61
Patient N. 4	0.82	N/A	0.86	1.65	1.55	1.75
Patient N. 5	4.33	4.24	6.08	5	N/A	6.38
Patient N. 6	1.49	N/A	N/A	1.33	1.28	N/A
Patient N. 7	1.55	0.77	0.8	N/A	N/A	0.85
Patient N. 8	1.61	1.73	N/A	1.92	3.28	4.69
Patient N. 9	1.4	1.66	1.37	1.32	1.08	1.02
Patient N. 10	0.64	0.47	0.52	0.58	0.48	0.61
Patient N. 11	1.89	1.75	1.95	1.95	N/A	N/A

**Table 4:** CRP levels at hospitalization and during the drug administration.

CRP Levels	Day of Hospitalization	Drug Administration Day 1	Drug Administration Day 2	Drug Administration Day 3	Drug Administration Day 4	Drug Administration Day 5
Patient N. 1	148.17	N/A	39.04	17.14	25.32	N/A
Patient N. 2	194.39	83.76	N/A	148.84	126.91	N/A
Patient N. 3	182.3	20.11	14.26	9.41	4.74	16.05
Patient N. 4	29.61	N/A	13.4	7.08	3.08	1.36
Patient N. 5	135.94	104.72	74.72	63.78	N/A	31.67
Patient N. 6	77.14	N/A	N/A	21.24	16.25	N/A
Patient N. 7	274.5	6.08	5.83	N/A	N/A	2.13
Patient N. 8	199.26	77.14	N/A	116.34	113.51	111.14
Patient N. 9	139.5	97	130.3	88	52.1	41.4
Patient N. 10	8.33	16.53	20.24	16.87	13.04	15.27
Patient N. 11	9	N/A	14.9	15.7	N/A	N/A

**Table 5:** WBC levels at hospitalization and during the drug administration.

WBC Levels	Day of Hospitalization	Drug Administration Day 1	Drug Administration Day 2	Drug Administration Day 3	Drug Administration Day 4	Drug Administration Day 5
Patient N. 1	9.1	8.1	14	13.3	13.7	12.5
Patient N. 2	2.6	2.1	N/A	3.3	3.4	N/A
Patient N. 3	13.9	9.6	10.3	10.5	9.2	12.6
Patient N. 4	4.6	N/A	9.9	11.3	13.2	18.2
Patient N. 5	1.8	2.6	3.9	3.9	N/A	6.7
Patient N. 6	9.8	N/A	N/A	6.7	7.6	N/A
Patient N. 7	7.8	9.4	10.4	N/A	N/A	9.6
Patient N. 8	5.9	8.3	N/A	10.2	15.7	18.1
Patient N. 9	12.5	16.1	9.9	8	10.4	8.6
Patient N. 10	2.8	3.1	2.7	4	10.9	11.7
Patient N. 11	5.5	N/A	N/A	15.6	N/A	N/A

## Data Availability

No individual data will be shared, only the de-identified data regarding medical history and clinical outcomes during the drug administration. Only researchers can access the data.
